# Clinical, Socioeconomic, and Demographic Factors Associated with Diabetic Retinopathy in a Hospital-Based Screening Program: A Cross-Sectional Study in Oslo, Norway

**DOI:** 10.3390/healthcare14182929

**Published:** 2026-09-09

**Authors:** Katrine Holen, Mia Karabeg, Ellen Steffenssen Sauesund, Dag Sigurd Fosmark, Marius Dalby, Beata Eva Petrovski, Goran Petrovski

**Affiliations:** 1 Department of Ophthalmology, Oslo University Hospital, 0450 Oslo, Norway; mia.karabeg@studmed.uio.no (M.K.); ellen.s.sauesund@gmail.com (E.S.S.); doktordag@icloud.com (D.S.F.); b.e.petrovski@medisin.uio.no (B.E.P.); goran.petrovski@medisin.uio.no (G.P.); 2 Center for Eye Research and Innovative Diagnostics, Department of Ophthalmology, Institute of Clinical Medicine, Faculty of Medicine, University of Oslo, 0450 Oslo, Norway; 3 Department of Ophthalmology, Vestre Viken Hospital Trust, 3004 Drammen, Norway; marius.dalby@gmail.com; 4 Lovisenberg Diaconal University College, 0456 Oslo, Norway; 5 Department of Ophthalmology, School of Medicine, University of Split, 21000 Split, Croatia; 6 UKLO Network, University St. Kliment Ohridski-Bitola, 7000 Bitola, North Macedonia

**Keywords:** diabetic retinopathy, diabetes mellitus, diabetic retinopathy screening, socioeconomic factors, risk factors, hemoglobin A1c (HbA1c)

## Abstract

**Background:** Diabetic retinopathy (DR) is a major cause of visual impairment. Its clinical determinants are well-established, whereas socioeconomic associations vary across settings. **Objective:** To estimate the observed proportion of DR and examine associations between clinical, demographic, and socioeconomic factors and DR among adults attending a hospital-based screening program in Oslo, Norway. **Methods:** This cross-sectional study analyzed 118 adults with diabetes. DR was graded from wide-field retinal images. A complete-case multivariable logistic regression model included age, diabetes duration, HbA1c, BMI, diabetes type, education level, and economic activity. **Results:** DR was present in 67 participants (56.8%). The adjusted model included 93 participants (53 with DR and 40 without DR). Longer diabetes duration was associated with higher odds of DR (adjusted odds ratio [aOR] 1.27 per year, 95% CI 1.14–1.41; *p* < 0.001), whereas age showed an inverse association (aOR 0.89 per year, 95% CI 0.82–0.97; *p* = 0.006). HbA1c, BMI, diabetes type, education, and economic activity were not statistically significant after adjustment. **Conclusions:** No statistically significant associations between the measured socioeconomic factors and DR were identified in this study sample. Clinically relevant associations and causal inference cannot be assumed because of possible selection bias and cross-sectional study design.

## 1. Introduction

Diabetic retinopathy (DR) is the foremost microvascular complication of diabetes mellitus (DM) [1] and a leading cause of preventable visual impairment and blindness in the working-age population of the industrialized world [1,2]. However, systematic screening [3,4] and advancements in technology and treatment [5,6] has changed this in many Western countries. These innovations have enhanced the early detection and treatment of DR, which are crucial to prevent severe vision loss [7,8].

The global prevalence of DR among people with DM is estimated to be approximately 35% [9], with substantial variability across regions [9,10,11,12]. Variations in the study designs, population demographics, and methods used to identify and classify DR have impeded the ability to make direct comparisons across studies [9]. Norwegian data, such as from the Tromsø Eye Study, have reported a DR prevalence of 26.8% and a prevalence of diabetic macular edema (DME) of 3.9% [13], while a pilot study for the implementation of DR screening in Oslo reported a DR prevalence of 27.8% [14], highlighting a substantial disease burden even in high-income regions.

Established clinical risk factors for developing DR include lifestyle and systemic vascular factors, such as hyperglycemia, Hemoglobin A1c (HbA1c), dyslipidemia, duration of diabetes, and hypertension [15]. However, the role of socioeconomic and demographic status in DR risk progression is less clearly defined, with previous studies yielding variable results [16,17,18,19]. More studies are therefore needed to explore the risk factors for the early detection of DR.

The global estimates from the International Diabetes Federation of almost 600 million adults (20–79 years of age) living with DM in 2024 [20] show a major and expanding health problem due to lifestyle and physical activity changes that contribute to obesity. Projections estimate the prevalence of DM to rise to 850 million by 2050 [20]. In Europe, there were 65.6 million individuals living with DM in 2024, with an expected increase to 72.4 million in 2050 [20]. In Norway alone, there is an estimated number of 316,000–345,000 individuals living with DM, including a substantial proportion of around 60,000 undiagnosed cases [21].

DM is considered a major public health problem and is an economic burden to healthcare systems worldwide that impacts people’s quality of life [22,23]. Understanding the incidence rates is crucial for the effective planning and allocation of healthcare services [24,25].

Screening programs employing retinal imaging, as recommended by the World Health Organization (WHO), are effective tools for early DR detection [26]. DR gives no or few symptoms until it reaches severe stages, such as the presence of proliferative diabetic retinopathy (PDR) and/or DME [27]. Screening for DR leads to the prevention of visual impairment in people with DM who are at high risk [27]. Several countries have implemented a national screening program to detect DR at an early stage [28,29]. In Scandinavia, Iceland, the country began DR screening in 1980 and was cited as a pioneer, while Sweden established regional DR screening in 1990 [3,30]. The Danish Registry of Diabetic Retinopathy (DiaBase) was established between 2003 and 2006 and was then extended nationwide in 2010 [31]. All countries have revealed a decrease in severe DR after implementing such programs. Norway has lagged in establishing a screening program, and comprehensive data on DR epidemiology and risk factors in a Norwegian registry remain incomplete. Understanding the clinical, socioeconomic, demographic, and behavioral determinants of DR is vital for optimizing screening strategies and preventing vision loss.

The aim of this study was to estimate the observed proportion of DR and to examine associations between clinical, demographic, and socioeconomic factors and the presence of DR among adults attending a hospital-based DR screening program in Oslo.

## 2. Patients and Methods

A cross-sectional study was conducted between March 2023 and December 2024 at the Department of Ophthalmology, Oslo University Hospital (OUH), on adult patients (≥18 years) with DM in accordance with the Declaration of Helsinki. The study was approved by the Regional Committee of Ethics in Medical and Health Research (REK nr.: 466341) and the Data Protection Officer (DPO) at OUH.

In the study period, 122 patients with a diagnosis of type 1 diabetes mellitus (T1D) or type 2 diabetes mellitus (T2D) were invited to participate upon first-time referral to a DR screening program using fundus photo acquisition at the outpatient clinic. Seven of the participants had previously been patients at the Department of Ophthalmology but had a new referral for the current matter. Patients referred from ophthalmologists and general practitioners under concession of the South-Eastern Norway Regional Health Authority were also invited.

Consenting participants completed validated self-report questionnaires capturing socioeconomic and demographic characteristics, medical history, anthropometrics, behavioral/lifestyle factors, physical activity, symptoms of sleep apnea, and quality of life. HbA1c values were obtained primarily from referrals or medical records. When no value was documented, participants reported their most recent HbA1c value. Clinical data were collected or documented by trained ophthalmic nurses.

Physical examinations included measurements of patients’ height; weight; waist, hip, and neck circumferences; BMI calculation; blood pressure; visual acuity; and intraocular pressure (IOP). Best corrected visual acuity (logMAR) and intraocular pressure (IOP) measurements for selected patients were performed by outpatient nurses.

The DR was graded according to the International Council of Ophthalmology (ICO) guidelines [32,33] using color fundus photography (OPTOS wide-field camera or CLARUS 700TM). Grading and quality control were performed by certified ophthalmology staff.

### Statistical Analysis

Descriptive statistics were used to summarize the data. Continuous variables were assessed using histograms, Q-Q plots, and Shapiro–Wilk tests and are reported as means with standard deviations or medians with interquartile ranges, as appropriate. Groups were compared using *t* tests, Mann–Whitney U tests, or chi-square tests. A single multivariable logistic regression model examined the associations with DR (no DR = 0; DR = 1). Statistical significance was set at *p* < 0.05.

The model included age (years), diabetes duration (years), HbA1c (mmol/mol), BMI (kg/m^2^), type of DM, education level, and economic activity. Age, diabetes duration, HbA1c, and BMI were modeled as continuous variables. The type of DM was modeled with T1D as the reference; education was represented by two indicator variables, with primary education as the reference; and economic activity was modeled with economically inactive individuals used as the reference. After the removal of missing values, complete-case analysis included 93 participants, of whom 53 had DR while 40 did not.

The outcome of the study was the presence of DR (DR = 1; no DR = 0). The complete-case model included 93 participants (53 with DR; 40 without DR). The results are shown as adjusted odds ratios (aORs) with 95% confidence intervals (CIs). T1D, primary education, and economically inactive individuals were used as the reference categories. Education was represented by two indicator variables.

Linearity in the logit was assessed for each continuous predictor by retaining the original term and adding an x × ln(x) term to the full model. There was no evidence of departure from linearity for age (*p* = 0.31), diabetes duration (*p* = 0.48), HbA1c (*p* = 0.65), or BMI (*p* = 0.68); therefore, these variables were retained as continuous. Variance inflation factors ranged from 1.11 to 3.48. Model diagnostics did not indicate a gross lack of fit, and the apparent area under the receiver operating characteristic curve was 0.92. Because performance was assessed in the development sample and the number of observations was limited relative to the eight predictor coefficients, these diagnostics were interpreted cautiously rather than as evidence of a validated model.

## 3. Results

**Figure 1:**
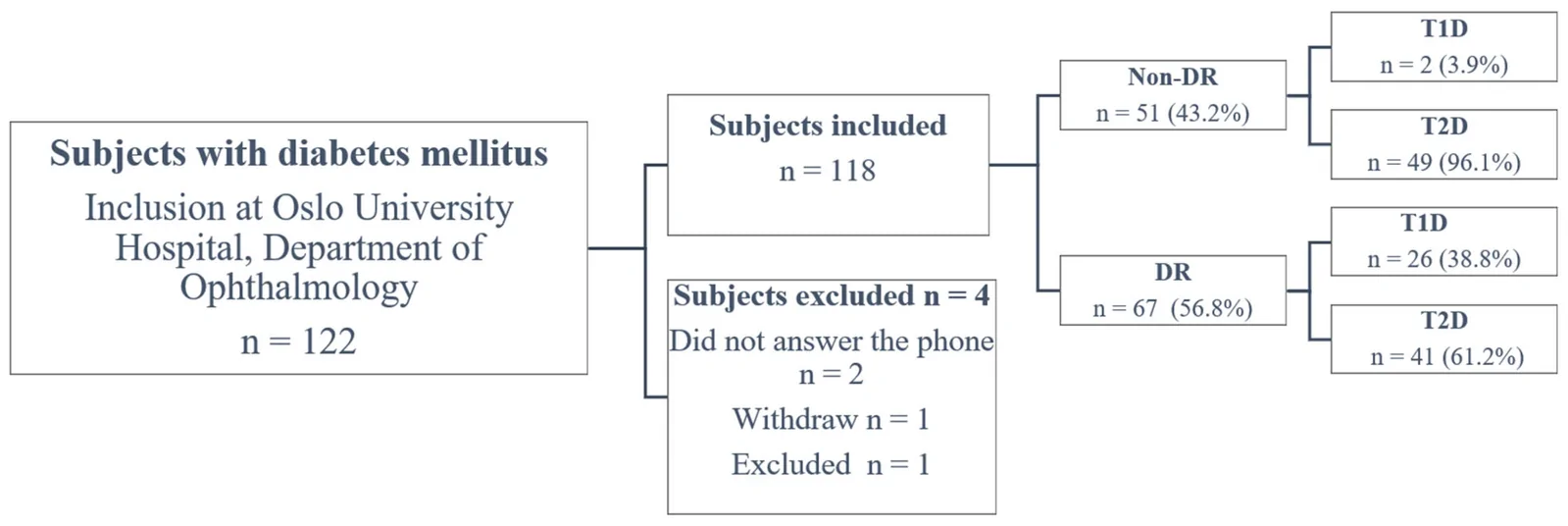
Flow chart of the study population. DR, diabetic retinopathy; n, number of cases; T1D and T2D, type 1 and 2 diabetes mellitus.

Figure 1 describes the study population. In total, 122 individuals were initially recruited into the study, out of whom four were excluded (one failed to meet the inclusion criteria; one withdrew; and two could not be reached for follow-up to complete the questionnaires).

Table 1 summarizes the demographic characteristics of the 118 participants. DR was present in 67 participants (56.8%), whereas 51 had no DR. Participants with DR were younger (50.7 ± 15.3 versus 58.5 ± 12.5 years; *p* = 0.002), and diabetes type differed between groups (*p* < 0.001). Gender and ethnicity did not differ significantly between groups.

Table 2 summarizes the clinical characteristics. In unadjusted comparisons, the BMI category differed between groups (*p* = 0.01), and participants with DR had a longer diabetes duration (median 18.5 versus 2.0 years; *p* < 0.001) and higher HbA1c (median 58.5 versus 51 mmol/mol; *p* = 0.003). These unadjusted comparisons should not be interpreted as independent adjusted associations.

Table 3 shows the socioeconomic characteristics of the study population. Higher education was most common in both DR groups (47.1% vs. 48.5%). The DR group had a higher proportion of economically inactive (participants not being in active employment at the time of the study) individuals (53.0% vs. 49.0%). Non-smokers were more common in the DR group (76.1% vs. 70.6%), and alcohol consumption was higher in the DR group (68.2% vs. 76.5%), though none of the differences were statistically significant.

Table 4 presents the adjusted associations. A longer diabetes duration was associated with higher odds of DR (aOR 1.27 per year, 95% CI 1.14–1.41; *p* < 0.001), whereas age showed an inverse association (aOR 0.89 per year, 95% CI 0.82–0.97; *p* = 0.006). HbA1c, BMI, type of DM, education level, and economic activity were not statistically significant. The confidence intervals for type of DM and education were wide, indicating considerable uncertainty in the magnitude and direction of these estimates.

## 4. Discussion

This hospital-based cross-sectional study examined clinical, demographic, and socioeconomic associations with DR among adults referred for screening in Oslo. In the adjusted model, a longer duration of DM was associated with higher odds of DR, whereas age showed an inverse association. HbA1c, BMI, type of DM, education, and economic activity were not statistically significant after adjustment. The study therefore primarily evaluates the established associations in a Norwegian screening setting instead of identifying new causal risk factors.

The observed DR proportion of 56.8% applies to this referred hospital-based sample and should not be interpreted as population prevalence. The inverse association with age persisted after adjustment for DM duration and type of DM and therefore cannot be explained solely by their distributions. Possible explanations include age at DM onset, referral patterns, selection into hospital screening, and residual confounding. The result must not be interpreted as evidence that older age protects against DR.

No statistically significant adjusted associations were identified for education or economic activity. This absence of statistical significance is not evidence that socioeconomic factors are unrelated to DR. The wide education confidence intervals—particularly for higher versus primary education (aOR 5.83, 95% CI 0.68–50.22)—show that the data are compatible with effects ranging from lower to substantially higher odds. This imprecision reflects the small reference subgroup, the modest complete-case sample, and the number of fitted parameters. Overall model diagnostics and coefficient precision address different questions: acceptable apparent calibration or discrimination does not make an individual coefficient precise. The absence of income data and possible attenuation of socioeconomic gradients within Norway’s comparatively equitable healthcare system are additional tentative explanations.

The duration of DM had the clearest adjusted association: each additional year was associated with 27% higher odds of DR (aOR 1.27, 95% CI 1.14–1.41). Age showed an inverse adjusted association that requires cautious interpretation. The estimates for BMI, HbA1c, type of DM, education, and economic activity were not statistically significant and should not be described as established associations. In particular, the wide intervals for type of DM and education indicate substantial uncertainty rather than evidence of no effect.

All participants had already been referred to a hospital-based fundus-photography service and therefore may not represent the broader Norwegian diabetes population. People who do not attend screening or have weaker contact with healthcare services may be underrepresented, potentially affecting both the observed DR proportion and the socioeconomic results. Questionnaire administration may have introduced a social-desirability bias. HbA1c was obtained primarily from referrals, medical records, or, if otherwise, by self-report; missing HbA1c values cannot be interpreted as poor self-management or non-adherence.

The modest sample size and small subgroups limited precision and the ability to identify smaller but clinically relevant associations. The complete-case model contained 93 participants and eight predictor coefficients; there were 53 DR events and 40 non-events. Consequently, overfitting and unstable estimates remain concerns despite apparently favorable in-sample discrimination. The model results should therefore be considered exploratory.

International studies, along with the research conducted in Scandinavia, confirm the paramount importance of organized screening in reducing sight-threatening DR, especially when participation is high and coverage equitable [3,31,34,35,36]. The findings of this cross-sectional study of adults with T1D and T2D in a DM screening program in Oslo, Norway, suggest that the Norwegian system has, at least in this sample, achieved relatively good access and outcomes irrespective of socioeconomic position, although ongoing vigilance and targeted outreach remain necessary [19].

Prior research has also shown that lower education and a history of smoking is associated with poor adherence to DR screening programs [37]. Although no such influence was seen here, this may reflect the specific population, methodology, or already-high population-level adherence to diabetes care recommendations in Norway [38]. As national guidelines and registry infrastructure for DR screening mature, they pave the way for continuous evaluation of participation across demographic and socioeconomic strata.

The high proportion of non-smokers and non-consumers of alcohol fits well with broader public health data from Norway [39], but it may also partially reflect reporting biases or the health-consciousness of those attending hospital-based screening. Systematic reviews stress that the combination of robust screening, clinical risk factor controls, and patient education—particularly around glycemic management—improves quality of life, reduces complications, and supports the economic sustainability of health systems [40,41].

The strengths of the study include standardized ophthalmic grading and the inclusion of clinical, demographic, and socioeconomic variables. The limitations of the study include its cross-sectional design, modest sample size, complete-case analysis, small exposure subgroups, possible model instability, self-reported information, absence of income data, and hospital-based selection. The design precludes causal inference. The observed DR proportion and socioeconomic findings should not be generalized to the overall Norwegian DM population.

Future studies should use larger, registry-linked Norwegian samples, enabling more granular evaluation of urban–rural differences, genetic risks, and health service interventions [31]. Additionally, the impact of new diabetes technologies and telemedicine on DR incidence and screening uptake should be explored.

## 5. Conclusions

This study on clinical, socioeconomic, and demographic factors associated with DR in a hospital-based screening program revealed that no statistically significant associations between the measured socioeconomic factors and DR were identified in this sample. However, clinically relevant associations cannot be excluded because of the modest sample size, limited precision, and possible selection bias. A longer duration of DM was associated with higher odds of DR, whereas the inverse age association requires cautious interpretation. Larger longitudinal, population-based studies are needed to assess these temporal and causal relationships.

## Figures and Tables

**Table 1:** Demographic and general characteristics of the study participants.

	Non-DR n = 51 (43.2%)	DR n = 67 (56.8%)	*p*-Value
**Gender**			0.8
Female	15 (29.4%)	21 (31.3%)	
Male	36 (70.6%)	46 (68.7%)	
**Type of diabetes**			**<0.001**
T1D	2 (3.9%)	26 (38.8%)	
T2D	49 (96.1%)	41 (61.2%)	
**Age** (years)			**0.002**
Mean ± SD	58.5 ± 12.5	50.7 ± 15.3	
**Ethnicity**			0.2
Caucasian	37 (72.6%)	51 (76.1%)	
Asian	7 (13.7%)	13 (19.4%)	
African	7 (13.7%)	3 (4.5%)	

*p* < 0.05 (in bold). DR, diabetes retinopathy; T1D and T2D, type 1 and 2 diabetes mellitus; n, number of cases; SD, standard deviation.

**Table 2:** Clinical characteristics of the study participants.

	Non-DR n = 51 (%)	DR n = 67 (%)	*p*-Value
**BMI**			**0.01**
Under/normal (≤24.9)	7 (14.0)	25 (37.9)	
Overweight (25.0–29.9)	21 (42.0)	24 (36.4)	
Obesity (≥30.0)	22 (44.0)	17 (25.8)	
**HbA1C** (mmol/mol)			**0.03**
Median (IQR)	51 (47–61)	58.5 (53–67)	
Range	37–108	39–106	
**Duration of DM** (years)			**<0.001**
Median (IQR)	2 (0.3–5)	18.5 (10–25)	
Range	0.1–23	0.1–60	
**Blood pressure**			0.6
Normal (≤140/90 mmHg)	43 (84.3)	54 (80.6)	
High (>140/90 mmHg)	8 (15.7)	13 (19.4)	
**Blood pressure medication**			0.4
No	24 (47.1)	37 (55.2)	
Yes	27 (52.9)	30 (44.8)	
**Cholesterol lowering** **medication**			0.8
No	21 (41.2)	29 (43.3)	
Yes	30 (58.8)	38 (56.8)	
**Dialysis**			0.5
No	51 (100.0)	64 (97.0)	
Yes	0 (100.0)	2 (3.0)	
**Other kidney disease**			0.2
No	50 (98.0)	61 (92.4)	
Yes	1 (2)	5 (7.6)	
**Myocardial infarction**			0.7
No	46 (90.2)	58 (87.9)	
Yes	5 (9.8)	8 (12.1)	
**Peripheral stent**			1.0
No	50 (98)	65 (98.5)	
Yes	1 (2)	1 (1.5)	
**Stroke**			1.0
No	48 (94.1)	62 (93.9)	
Yes	3 (5.9)	4 (6.1)	

*p* < 0.05 (in bold). IQR, interquartile range; n, number of cases; DR, diabetes retinopathy; DM, diabetes mellitus.

**Table 3:** Socioeconomic characteristics of the study population.

	Non-DR n = 51 (%)	DR n = 67 (%)	*p*-Value
**Education level**			1.0
Primary	6 (11.8)	8 (12.1)	
Secondary	21 (41.2)	26 (39.4)	
Higher	24 (47.1)	32 (48.5)	
**Economic activity status**			0.7
Economically inactive	25 (49.0)	35 (53.0)	
Economically active	26 (51.0)	31 (47.0)	
**Smoking**			0.4
No	36 (70.6)	51 (76.1)	
Yes	15 (29.4)	16 (23.9)	
**Alcohol**			0.3
No	12 (23.5)	21 (31.82)	
Yes	39 (76.5)	45 (68.2)	

n: number of cases; DR: diabetes retinopathy.

**Table 4:** Multivariable logistic regression model for the presence of diabetic retinopathy.

Variable	aOR	95% CI	*p* Value
Age, per year	0.89	0.82–0.97	0.006
Duration of diabetes, per year	1.27	1.14–1.41	<0.001
HbA1c, per mmol/mol	1.000	0.96–1.04	1.0
BMI, per kg/m^2^	0.92	0.81–1.05	0.2
Diabetes type: T2D vs. T1D	2.03	0.19–21.40	0.6
Education: Secondary vs. primary	1.99	0.24–16.43	0.5
Education: Higher vs. primary	5.83	0.68–50.22	0.1
Economically active vs. inactive	0.31	0.07–1.42	0.1

T1D and T2D: type 1 and 2 diabetes mellitus.

## Data Availability

The datasets generated and/or analyzed during the current study are not publicly available because they contain sensitive patient information and are subject to Norwegian data protection regulations. De-identified data may be made available from the corresponding author upon reasonable request and with permission from Oslo University Hospital and the relevant ethical authorities.
